# Observation of the Effect of Aging on the Structural Changes of Polyurethane/Polyurea Coatings

**DOI:** 10.3390/polym16010023

**Published:** 2023-12-20

**Authors:** Paulina Mayer-Trzaskowska, Mariola Robakowska, Łukasz Gierz, Joanna Pach, Ewa Mazur

**Affiliations:** 1 Department of Light Elements Engineering, Faculty of Mechanical Engineering, Wroclaw University of Science and Technology, Foundry and Automation, Wybrzeże Wyspiańskiego 27, 50-370 Wroclaw, Poland; paulina.mayer@pwr.edu.pl (P.M.-T.); joanna.pach@pwr.edu.pl (J.P.); ewa.mazur94@gmail.com (E.M.); 2 Department of Polymer, Faculty of Chemical Technology, Poznan University of Technology, Berdychowo 4, 60-965 Poznan, Poland; mariola.robakowska@put.poznan.pl; 3 Faculty of Mechanical Engineering, Institute of Machine Design, Poznan University of Technology, Piotrowo 3, 60-965 Poznan, Poland

**Keywords:** aging, adhesion, pull-off test, atmospheric aging, polyurea/polyurethane coatings, surface wettability, thermal properties

## Abstract

In this study, polyurethane/polyurea coatings were exposed to four different types of aging: aging in the natural environment (NC), accelerated ultraviolet aging (UV), aging in a sodium chloride solution (NaCl), and thermal aging (TC). To monitor the changes that occurred during these processes, the thickness was measured and microscopic and macroscopic observations were conducted continuously. The samples aged under various conditions were then subjected to spectroscopic (FTIR) and thermal (TGA) analysis to determine the possible structural changes in the coatings. The dependence of aging conditions on the physicochemical properties of the tested coating surfaces was thoroughly investigated and characterized by the water contact angle and surface free energy. Adhesion tests were performed after the established aging period. The results show that different aging conditions have a tremendous effect on the appearance and pull-off strength. The coatings that age in a NaCl solution are more affected in terms of their adhesion.

## 1. Introduction

Every technical and non-technical object is exposed to the destructive impact of its surrounding environment over its lifetime. Depending on the function, role, and location of the object, the conditions of these surroundings may vary. One of the most common methods to protect objects from the degenerative effects of the environment is the use of protective organic coatings [1,2]. As the environmental awareness of industries and customers increases, the need to extend the life of coatings grows with it. Ensuring the durability of coatings is important for protecting the environment and is essential for specific industries, such as the aircraft [3] or aerospace industries [4]. Epoxy coatings are mainly used as anticorrosion protection [5,6]. The adhesion properties of the corrosion protection coating systems used in offshore wind power plants have been the subject of research [7].

Polyurethanes and polyureas are polymers frequently used to manufacture protective coatings, and in addition to their anticorrosive properties, they are often used as waterproofing materials, but also as protection against air-blast loading [8,9]. The difference between polyurea and polyurethane is the type of functional group involved in the reaction with isocyanates. Both are based on a two-component system, and the curing mechanisms are by reaction, where isocyanate is a common component, and the second component is a polyether polyamine for polyureas, while polyether polyol is for polyurethane [10]. It is also possible to make so-called ‘hybrid’ systems, in which the isocyanate reacts with a mixture of hydroxyl and amino groups [10]. A hybrid reaction requiring the use of catalysts becomes uncontrolled when the application takes place within ambient conditions relative to the recommended standards. At lower temperatures, the cross-linking of bonds may occur less frequently, while at higher temperatures, it is denser, which affects the mechanical properties of the finished product. Although the properties of polyurea are slightly better, hybrid coatings can replace them completely if less demanding components are insulated. However, hybrid materials are cheaper than pure polyurea.

Depending on the needs, these coatings can be applied manually or using spray units, which gives a wide range of application possibilities. However, adhesion to the substrate and other application properties, such as gloss, thermal stability, and contact angle, are very important. The correct adhesion affects the usability and determines the effectiveness of the applied coatings. There are many methods to determine the adhesion of the coating to the substrate. In the papers written by Vaca-Cortes and others, methods such as pull-off, peel, tear, knife, salt spray, hot water immersion, and cathodic disbondment are described [11,12,13]. One of the most commonly used methods to measure the force of detachment of the coating from the substrate is the destructive pull-off method [14,15].

The key factors that affect the adhesion of a polymer coating to a metal substrate are the proper preparation of the substrate, the chemical modification of the polymer, the use of intermediate layers, and the technological parameters of the coating process [16]. Methods to prepare the substrate surface have a powerful effect on the pull-off strength of the coatings [17]. One of the most effective methods for cleaning and improving the roughness of a steel substrate is abrasive blasting [18,19]. Physical pretreatment methods (plasma or vacuum UV) also increase the adhesion force on polymers [20].

As we know, coatings are often affected by aggressive factors, such as ultraviolet radiation, aggressive chemicals, mechanical stress, moisture, etc., which may damage the coating. Based on this background, research on the physicochemical and thermal properties of polyureas and polyurethanes is becoming more and more popular [21,22].

The service life may be reduced as a result of a weak adhesive connection between the coating and the substrate. Adhesion can decrease due to degenerative factors such as sunlight, temperature, water, chemical compounds, bacteria, and stress [23,24]. For this reason, it is essential to study the degradation mechanism of various coating materials in different types of environments [25]. Since aging in natural surroundings takes many years, researchers rely primarily on accelerated aging tests [26,27,28,29,30,31]. Unfortunately, it is impossible to truly predict the lifetime of different coating materials, as accelerated tests cannot account for every variable and because outdoor environments are so variable. There are many scientific papers available in which the authors conducted aging tests on polymer coatings applied to various surfaces (e.g., steel, wood, or concrete). For example, [32] presents the results of accelerated UV aging on an alkyd coating and a polyurethane coating. The results revealed that the alkyd system was less water resistant than the polyurethane, which was also confirmed by various mechanisms of destruction. Zhang [33] conducted comparative studies for typical marine coating systems after natural exposure and an artificial aging test. In the course of extended testing, micropores and cracks appeared gradually on the surface of the polyurethane paints, while the gloss of the coating was also diminished. The authors of [34] studied different spray paints that were exposed to artificial daylight or UV-A/B or C radiation. Che et al. [35] investigated the aging behavior and mechanism of polyurea coating in a marine atmosphere.

This paper provides an analysis of surface changes, physicochemical properties, thermal properties, and adhesion on polyurea/polyurethane coatings (applied in different ways) after exposure to natural weather conditions, ultraviolet (UV) radiation, immersion in a NaCl solution, and changing temperatures. Changes in surface morphology were monitored during the study. The tests revealed considerable differences in the pull-off strength depending on the aging conditions of the coatings. The samples aged under different conditions were subjected to spectroscopic (FTIR) and thermal analysis (TGA) to determine any structural changes in the coatings. The effect of aging conditions on the physicochemical properties of the coatings’ surface was thoroughly investigated and characterized by the water contact angle and surface free energy. The main goal of this investigation is to better understand the relationship between coating deterioration and protective performance by conducting adhesion tests and to help provide a comprehensive method for evaluating coating materials in steel under different aging conditions.

New about this work is the comparison of the adhesive properties of polyurea–polyurethane coatings used, among others, such as hydro-insulation, which are applied using various methods. Almacoat Floor SL is a coating that is poured or applied manually using rollers or brushes, while Almacoat Protect C is obtained by pneumatic spraying using special guns or reactors. These coatings were subjected to aging tests that reproduce the conditions prevailing in the natural environment of use of these coatings. The obtained results have utilitarian (practical) significance.

## 2. Materials and Methods

### 2.1. Materials

A 1.0 mm thick DC01 steel sheet was chosen as the substrate (EN 10130, chemical composition: Mn—0.60%, C—0.12%, P—0.045%, S—0.045%, Fe—remainder). This steel is a low carbon, cold-rolled structural steel intended for cold forming. Due to its high shapeability, it is used in the automotive, electronics, and household appliances industries. The steel sheet was cut into 14.5 mm × 3.5 mm samples.

The surface of all the prepared samples was mechanically prepared using the abrasive blasting method. The abrasive was corundum with a grain size of F30 [600–710 µm]. The surface was degreased with acetone. After the substrate was prepared, the surface roughness was measured. This test was carried out with the contact method using a TR100 surface roughness profilometer (Beijing TIME High Technology Ltd., Beijing, China). The surface roughness was determined from the mean elevation deviation of the profile Ra and the maximum height of the profile Rz. Ra was 3.10 ± 0.38 µm, and Rz was 26.42 ± 4.67.

**Figure 1:**
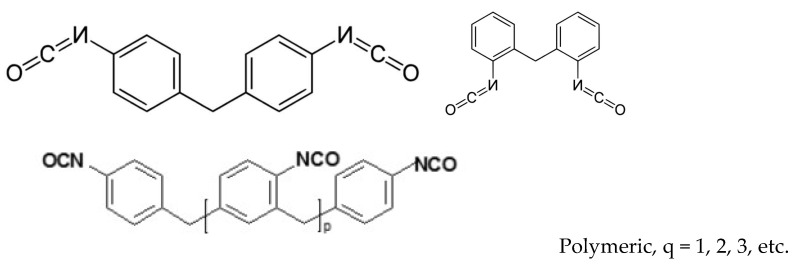
Ingredients of composition A.

All coating materials were applied according to the manufacturer’s recommendations. Almacoat Floor SL polyurea/polyurethane (Alma-color Sp. z o.o., Gniew, Poland) was applied manually by brush. Almacoat Protect C polyurethane/polyurea (Alma-color Sp. z o.o.) was applied by air spray using a MixCoat pneumatic gun (Sulzer, Tokyo, Japan) at 23 °C with a pressure of 0.65 MPa. Almacoat Floor SL and Protect C are two-component solvent-free coatings used on various surfaces: wood, concrete, steel, and aluminum. The composition of the resins is the manufacturer’s secret; the composition is very generalized. The polyol component is not indicated at all, although it is present in the composition. According to the manufacturer’s declaration, in both systems, component A consists of a diphenylmethane diisocyanate prepolymer (40–60%), methylene diphenyl diisocyanate (10–30%), and methylene diphenyl diisocyanate (5–15%). The general patterns are shown in Figure 1.

Components B of the Almacoat Floor SL system (Alma-Color Sp. z o.o., Gniew, Poland) include monomers based on polyoxypropylenediamine (40–60%), diethylmethylbenzenediamine (20–30%), and titanium dioxide (0–2%). It contains zero volatile organic content, which is extremely friendly to the environment.

After application, all samples were naturally cured in air at room temperature for 7 days. Table 1 summarizes the data on the resins used in the tests. The thickness of the coatings was measured in accordance with [36] with a coating thickness gauge that uses magnetic induction: a MiniTest 730/Sensor FN 5 (ElektroPhysik, Köln, Germany). The average thickness of the AFSL coatings was 250 ± 45 µm, while the APC coatings were 330 ± 30 µm thick.

### 2.2. Aging Conditions

Due to the fact that the aging process is influenced by many factors, it was decided to implement four different aging methods in the study, consisting of exposing the coatings to the following:

- Natural conditions (NC);
- UV radiation (UV);
- A NaCl aqueous solution (NaCl);
- Temperatures changes (TC).

**Figure 2:**
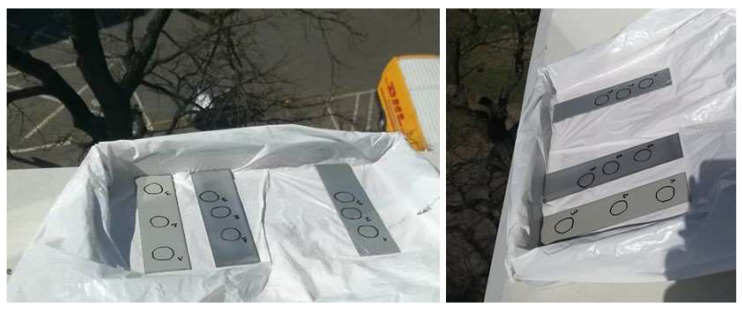
Aging process under natural conditions.

Natural weathering tests were performed in Wrocław, Poland, where the climate is temperate. The tests lasted five weeks and were conducted in spring (April/May 2022). The average temperature was 17.5 °C during the day and 7 °C at night. Of thirty-five days of exposure, seven days were rainy, and the others were sunny or partly cloudy. The samples were exposed at a 45° angle facing west (Figure 2).

Some samples were exposed to UV light in a chamber equipped with two 18 W and two 36 W fluorescent lamps (T8), which emit UV-A radiation at wavelengths between 350 and 400 nm. Accelerated UV exposure was carried out for five weeks.

To simulate a marine environment, some samples were exposed to a 5% NaCl solution. The compound was made by dissolving sodium chloride in distilled water. The samples were completely immersed in the solution for five weeks at room temperature.

Thermal aging was conducted periodically. A single cycle consisted of 2 h at −18 °C and 1 h at 60 °C. In total, the samples were subjected to 20 cycles with an average break of 24 h between cycles.

To determine the impact of aging on the coatings, measurements of the thickness of the coatings were taken. For samples aged under natural conditions, tests were carried out under a UV lamp and in NaCl solution after each week of aging, while for samples subjected to variable temperatures, tests were carried out after every five cycles.

### 2.3. Microscopic and Macroscopic Observation

To determine the impact of aging on a given film-forming material, the surface condition was assessed with microscopic photos. For samples aged under natural conditions, tests were carried out under a UV lamp and in NaCl solution after each week of aging, while for samples subjected to variable temperatures, tests were carried out after every five cycles. The surface condition of the coatings was observed using an Optatech stereoscopic microscope at 45× magnification. Changes were also visually assessed on a macroscopic scale.

### 2.4. Gloss Measurement

Gloss measurements were made in accordance with the [37] using a specular gloss meter. The measurement angle was 60° (measuring range: 0 to 300 GU) and the measurement area was 9 × 15 mm. Gloss was determined by directing a beam of light with a constant intensity and at a specified angle onto a surface and measuring the amount of light reflected at the opposite angle. The gloss value is influenced by the properties of the material, such as its curvature, the thickness of the film, the unevenness of the surface finish, the orientation of the sample, and others [38].

### 2.5. Surface Wetting

The water contact angle in the coatings was measured by the sessile drop method using an OCA 15EC contact angle goniometer with an accuracy of ±0.01 mN/m (DataPhysics Instruments GmbH, Filderstadt, Germany) at room temperature (approximately 25 °C). Water droplets of 0.2 μL were deposited at ten random locations in the coatings for each measurement. The images of the droplets were recorded and analyzed using the SCA 20 shape analysis software, 2017 [39] (DataPhysics Instruments GmbH, Filderstadt, Germany).

The surface free energy (SFE) of the coatings and their components was determined based on the Owens-Wendt-Rabel-Kaelble (OWRK) method [40]. In the OWRK method, the surface free energy of a solid is calculated from the contact angle created by at least two liquids placed on the surface of the solid, where the polar and dispersive components are known. SFE is the sum of the polar (γsp) and dispersive (γsd) components (1). The polar and dispersive components of the coatings under study were calculated by the Instrument Software [40]. As test liquids, water and PEG 200 (polyethylene glycol) were used.

SFE = γsp + γsd(1)

### 2.6. Pull-Off Adhesion Test

**Figure 3:**
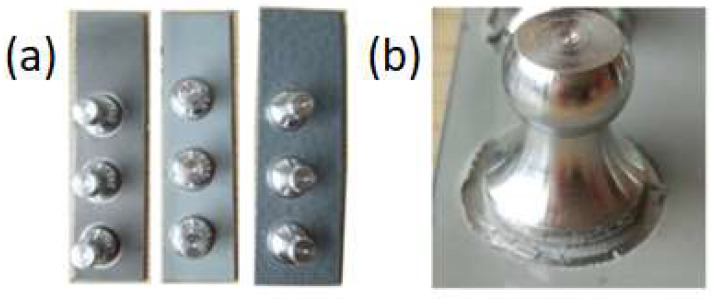
Photos from the pull-off strength tests showing (**a**) samples of coatings with measuring dollies glued in place and (**b**) the coating around a measuring dolly being cut out.

The polyurea coating pull-off adhesion test was carried out on different polymer substrates and was carried out according to [41] with a Posi Test AT pull-off adhesion tester, which can determine the greatest tensile pull-off force that a coating can support before detaching. Breaking points, demonstrated by fractures on the surface, occur along the weakest plane within a system consisting of a dolly, glue, the coating layers, and the substrate. When the pull-off test was performed, the dolly and coated surface were examined. A methacrylic adhesive was used to glue five aluminum dollies (20 mm in diameter) to each sample. After 24 h, a circular notch was cut around the stamp and the pull test could be performed. Samples prepared for measurement are presented in Figure 3.

### 2.7. Thermal Properties

Thermal resistance was investigated with a Tarsus thermogravimetric analyzer TG 209 F3 (Netzsch-Geratebau GmbH, Filderstadt, Germany). Samples weighing 10 mg were heated in Al_2_O_3_ crucibles from 40 to 700 °C at a scan rate of 10 °C/min in a nitrogen atmosphere (a 20 mL/min of N_2_ protection gas and 30 mL/min purge of N_2_ sample gas). The decomposition temperature of the material was measured at 10%, 50%, and 90% weight loss. The residual mass (R) was defined at about 600 °C.

### 2.8. FTIR Spectroscopy

Fourier transform infrared (FTIR) spectra of the products were obtained using the attenuated total reflectance (ATR) mode of a Nicolet 5700 spectrometer equipped with a ZnSe crystal ATR unit (Thermo Fisher Scientific Inc., Branchburg, NJ, USA) and a Bruker (Billeta, MA, USA) Tensor 27 equipped with a SPECAC Golden Gate diamond ATR accessory (Bruker Optik GmbH, Ettlingen, Germany). The spectra were recorded with a resolution of 4 cm^−1^ and an accumulation of 64 spectra.

The FTIR-ATR absorption spectra were used to calculate the photo-oxidation index (PI) and the carbonyl index (CI), as in Equation (2) [42]:

(2) $$Cl=\frac{A_{C=O}}{A_{CH_{3}}}$$

where A_c=o_ is the area of the carbonyl absorption band and A_CH3_ is the area of the absorption band attributed to the C–H band.

## 3. Results and Discussion

### 3.1. Morphological Observation

**Figure 4:**
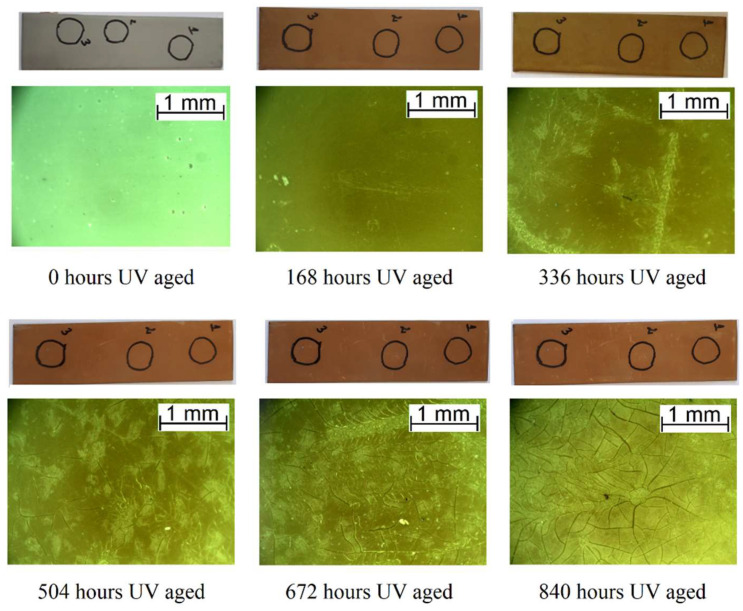
Macroscopic and microscopic images of the surface degradation of the AFSL polyurea/polyurethane coating during UV aging.

**Figure 5:**
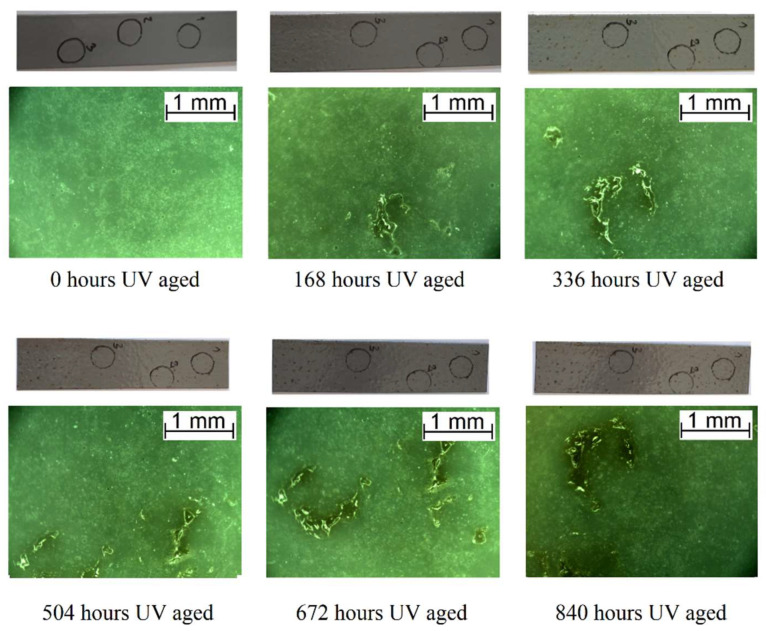
Macroscopic and microscopic images of the surface degradation of the APC polyurethane/polyurea coating during UV aging.

Morphological observation was carried out for all the samples after each week of the aging process. The most noticeable changes occurred during accelerated ultraviolet aging. UV radiation had a major impact on the morphology of the AFSL polyurea/polyurethane coating (Figure 4). Even after the first week of aging, the coating was visibly yellowing and defects were noticed on the surface. The color change is generally attributed to photodegradation, caused by the formation of free radicals due to chromophoric groups in the polyurea structure that absorb UV radiation [43]. As the aging progressed, the coating lost its gloss and turned brown. Defects in the form of cavities became more numerous with longer aging and the microcracks propagating from them became progressively more extensive and visible. During UV aging of the APC polyurethane/polyurea, its color changed as well, but differently: brown spots formed on the surface that increased in size and quantity with longer aging. The marks were visible under the microscope as point defects (Figure 5).

The surface of the samples aged in a natural environment appeared similar to that of the samples aged by UV radiation. When the microstructures of the coatings were compared, it was observed that the degradation occurred in a similar way but to a lesser extent with UV radiation. The differences between the results obtained from these two aging methods may be related to the fact that UV-A radiation is not the main destructive factor of polymer coatings. Under natural conditions, polymers are exposed to many variable factors and the combined action of these factors has a greater impact on the properties than the UV radiation itself. Therefore, it was found that accelerated aging methods that take into account only the radiation factor are insufficient for predicting the durability of polymer coatings.

**Figure 6:**
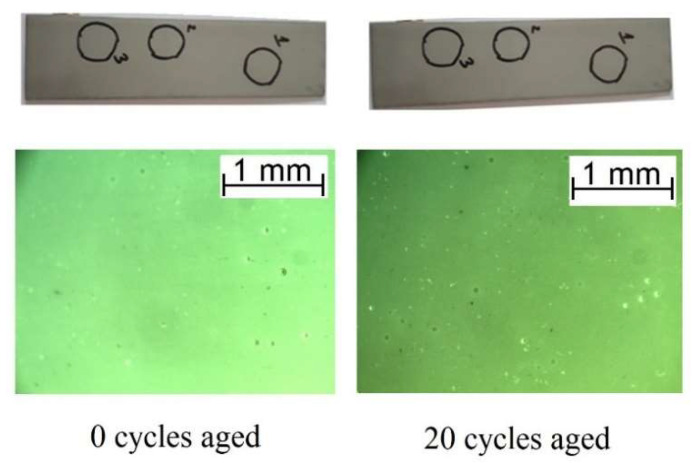
Macroscopic and microscopic images of the surface degradation of the AFSL polyurea/polyurethane coating before and after fluctuating temperatures.

**Figure 7:**
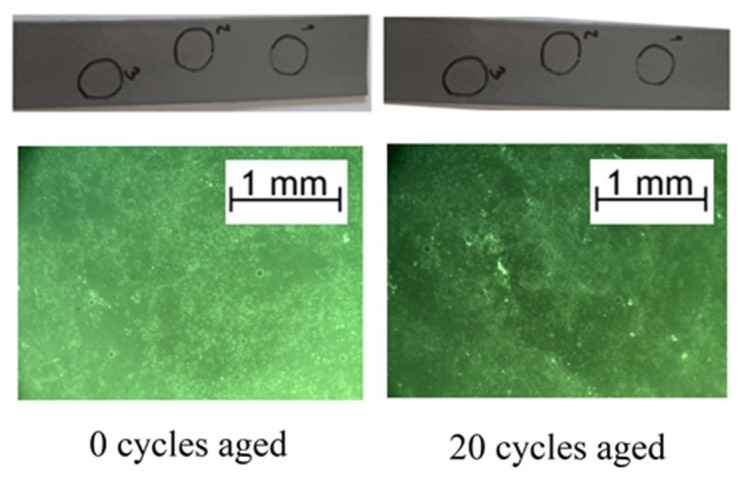
Macroscopic and microscopic images of the surface degradation of the APC polyurea/polyurethane coating before and after fluctuating temperatures.

Macroscopic and microscopic observations of the samples aged in sodium chloride solution and with thermal aging did not show any visible transformations (Figure 6 and Figure 7).

### 3.2. Gloss and Surface Wettability

**Figure 8:**
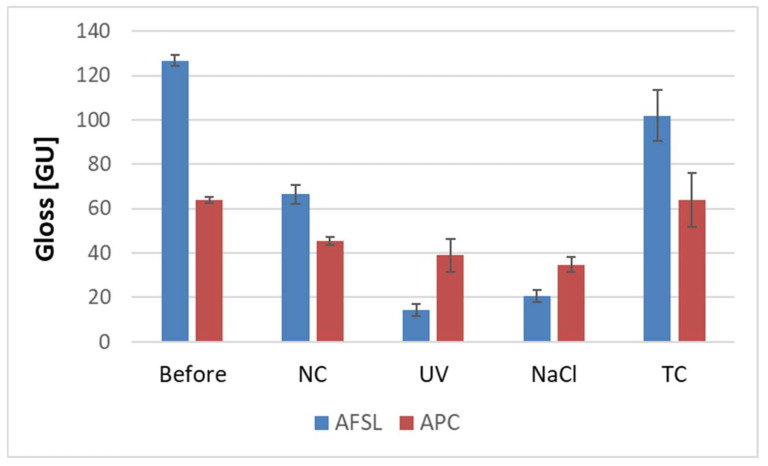
Gloss measurements of the coatings under study.

Gloss refers to the ability of a surface to reflect light projected onto it. The property can be used to indicate the degree of the aging of coatings. Figure 8 shows the changes in the gloss of polyurea/polyurethane coatings. The gloss of the APC coating was the lowest because the surface of this coating was the roughest; this was due to the spray method of its application. However, the AFSL coating, which was applied manually, had a smooth surface. The literature [33] states that the greater the roughness of the coating surface, the lower its reflectivity and gloss values; thus, the decreased gloss corresponded well to the cracks in the coating surface. A minor change in gloss was observed in the coatings subjected to fluctuating temperatures. With extended exposure time, the gloss value showed a decreasing tendency, especially for samples that were aged with ultraviolet light. The greatest decrease in gloss was observed for the AFSL polyurea/polyurethane coating after UV and NaCl aging, which was confirmed by microscopy with numerous microcracks and the greatest color change.

The various aging processes changed not only the morphology of the coatings but also their surface wettability. Surface wettability plays an essential role in many processes and material applications. Determining the angle of contact with water (θW) can inform about changes in surface morphology through the wetting properties and the hydrophilic or hydrophobic character of a coating surface [35]. The contact angles and surface energies of the polyurea/polyurethane coatings are presented in Table 2 and Table 3.

The contact angles of the AFSL and APC coatings before aging were approximately 82° and 83°, respectively. The greater the contact angle, the stronger the polar interactions are. When a solid surface is hydrophilic, the contact angle will be lower than 90°, whereas a hydrophobic material has a water contact angle greater than 90°. For AFSL-based polyurethane/polyurea coatings, the trend for contact angles was the same as that for gloss. The lowest contact angles were found for coatings aged with UV and NaCl; in this case, the drop decreased very quickly over time. This finding indicates a significant change in the wettability of a coating, which is associated with a decrease in its gloss. This aging also directly affects the surface properties of the coating being manufactured, especially its polar and dispersive components. Compared to unaged coatings, the contact angle was half as large and the surface energy was three times as high. As can be seen in Table 2 and Table 3, the polar components were predominant, which means that this coating has a high affinity for water.

In the case of the AFSL coatings aged with UV light or treated with NaCl, there was a large reduction in the wetting angle. This indicates a significant change in the wettability of the sample; as the value decreased, the level of hydrophilicity increased, which could increase the adhesion of contaminants [44]. In this case, SFE increased rapidly and polar interactions (i.e., polar hydrogen bond) were quite high, while the dispersive interaction was very small. In all samples of AFSL coatings, the dispersion component represented a much smaller proportion of the total SEP value than the polar component, whereas the opposite was observed in APC coatings (with the exception of TC-aged coatings).

### 3.3. Adhesion of Coatings

**Figure 9:**
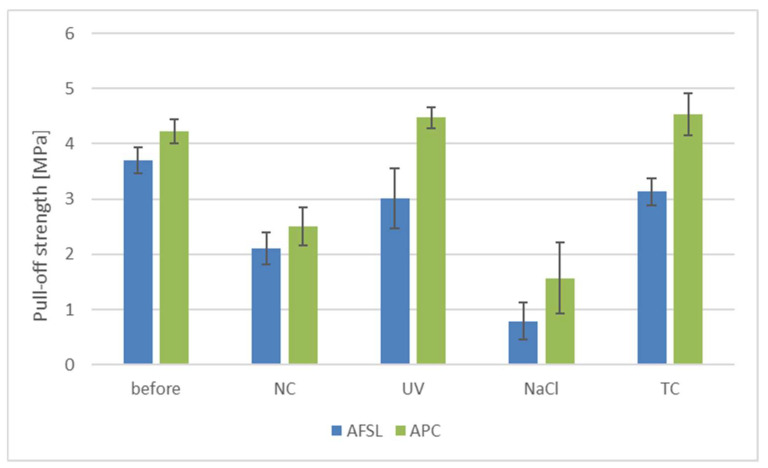
Pull-off strength of the tested coatings.

The adhesion measurements for every coating before and after aging are presented in Figure 9. The highest pull-off strength values (above 4 MPa) before aging were obtained for the APC polyurea/polyurethane coatings. Surprisingly, there was an increase in pull-off strength after this coating was subjected to variable temperature cycles. Placing the coating in an oven, where the temperature was 60 °C, could have contributed to the post-hardening of the coating, increasing its adhesion to the steel substrate. A similar effect occurred after UV aging of the increase in the same coatings, where the temperature also caused cross-linking of the polyurethane [45].

The aging of the coatings in NaCl solution had the greatest impact on their adhesion. Pull-off strength decreased four times for each of the tested coatings. The tests were carried out in an aqueous environment, where the samples were completely immersed in the solution for five weeks, resulting in the liquid penetrating between the coating and the substrate. Research by scientists from China also confirmed the destructive impact of the aqueous environment on the appearance and properties of coatings applied to mortar substrates [46]. Comparative tests of epoxy and polyurea coatings showed that epoxy coatings degraded to a greater extent under the influence of UV aging. However, resistance to chlorides decreased significantly for polyurea coatings, resulting in poorer adhesion and waterproofness [47]. Natural aging had a greater effect on the adhesion of the coatings than the influence of ultraviolet (UV) radiation. The samples left in the natural environment were exposed to rain, which can also alter adhesion.

APC coatings sprayed with a gun have higher pull-off strength values. The authors of the publication [48] showed that even the parameters of the spraying process influenced the mechanical properties of polyurea coatings.

### 3.4. FTIR Spectroscopy

**Figure 10:**
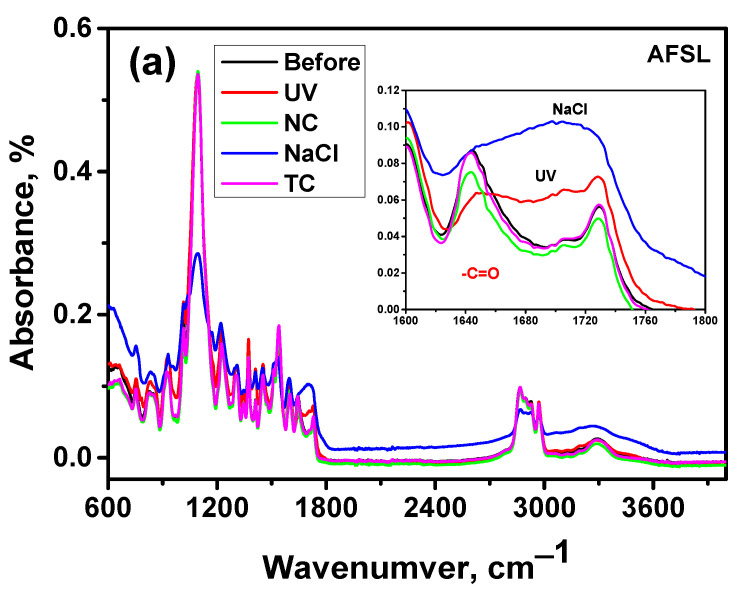

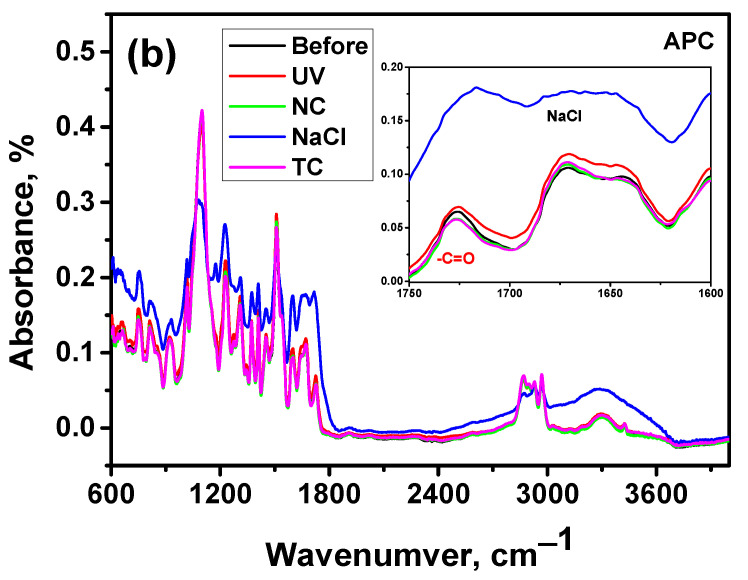
Experimental infrared absorption spectra of polyurea/polyurethane coatings: (**a**) APC and (**b**) AFSL before and after aging.

**Figure 11:**
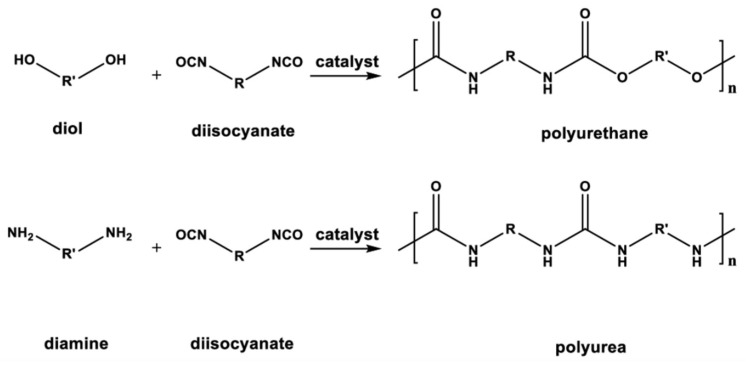
General reactions occurring in polymer coatings.

Infrared spectroscopy was used to assess structural changes in unaged coatings and those aged by different aging processes (Figure 10). The main bands of the FTIR spectra can be assigned according to the published data. From a chemical standpoint, polyurethane is made from the reaction of an isocyanate with a polyol, whereas polyurea is formed from an isocyanate reacting with a multifunctional amine. The polyurea reaction is much faster than the polyurethane one (Figure 11). The isocyanate in this case exists in two forms: aromatic and aliphatic. In addition, this compound can act as a monomer, a polymer, or a mixture thereof [10]. It is the manufacturer’s secret which polyol compound was added to system B. According to the manufacturer’s declaration, in formulations, the resin is a homogeneous blend of polyetheramine characterized by repeating oxypropylene units. The hydrogen compound may be one of the reactants that replaces some long-chain polyether amines and reacts with isocyanate for the preparation of a polyurethane–urea copolymer. Urea groups are typically detected by FTIR because the vibration of the carbonyl bond in urea is significantly different from the vibration of the carbonyl bond in urethane groups [49]. As expected, vibrations from carbonyl groups of urea at 1634 cm^−1^ and for –NH bending at 1548 cm^−1^ were observed for polyurea, while vibrations associated with carbonyl groups of urethane groups at 1732 cm^−1^, 1600 cm^−1^ for the –NH bending, ~1510 cm^−1^ for amide II band, and 1066 cm^−1^ for C-O bonds were detected (Figure 9). A band observed at a wave number of ~1643 cm^−1^ matched the –C=O of a urea group that formed a hydrogen bond with the =NH group of another urea moiety in proximity. The absorption bands that formed around 1673–1685 cm^−1^ corresponded to free hydrogen-bonded urea carbonyl [35]. In the IR polyurea/polyurethane coatings, no bands appeared on a wave number of ~2270 cm^−1^ coming from the stretching of the –N=C=O group, indicating a complete polyaddition reaction.

The FTIR spectra show absorption bands at a wave number of 3350 cm^−1^, corresponding to stretching vibrations of N-H bonds; bands at about 2840 and 2960 cm^−1^, which are associated with symmetric and asymmetric vibrations of the C-H bonds of CH_2_ groups; and bands of deformational vibrations of the C-H bonds of CH_2_ groups, at about 1227 cm^−1^. In all cases, the maxima were also observed at a wave number of about 1100 cm^−1^, which confirms the presence of C-O bonds in the samples. Furthermore, polyetheramine absorption bands were present, with symmetric CH_2_-O-CH_2_ stretching at 921 cm^−1^, asymmetric CH_2_-O-CH_2_ stretching at 1100 cm^−1^, and C-N stretching at 1017 cm^−1^.

The FTIR spectra showed a very strong absorption peak at 1093 cm^−1^, which is attributed to the attached isocyanate CN stretch alkyl amine group. The aromatic primary amine in the AFSL coating, the CN stretch group, expressed itself at a wave number of 1345 cm^−1^. FTIR analysis confirmed the presence of both polyurethane and polyurea groups in the coatings. The characteristic bands of TiO_2_ appear on the FTIR spectrum, at wavenumbers of 600–700 cm^−1^, and are associated with the occurrence of Ti-O-Ti bond stretching vibrations. The broad range of the small peak (APC) from 1800~2200 cm^−1^ is attributed to the bending vibrations of Ti-O.

The spectra of UV-aged coatings (AFSL) and NaCl-aged coatings (AFSL and APC) showed important changes in the carbonyl stretching region, accompanied by more subtle changes observed at other wave numbers. The band in the carbonyl region can be assigned to hydrogen-bonded carbonyl vibration (NH-CO-NH of polyurea) or free carbonyl stretching (free C=O band at 1732 cm^−1^ and H-bonded C=O band at 1704 cm^−1^). After the mixture was aged, the carbonyl stretching vibration of the coatings appeared as a complex absorption band consisting of overlapping bands. In this case, a new wide band also appeared at 1770 cm^−1^, characterizing peroxide structures as a result of oxidation [43]. This broad band also suggests the formation of new carbonyl species, for example, quinine-imide structures (yellow), and the reduced peak at 1735 cm^−1^ corresponds to hydrolysis of the ester bond in the urethane bond [50].

Moreover, the coating spectra indicated a major change in the structure, especially of the coatings aged in NaCl. There was a significant broadening of the band in the range from 3000 to 3700 cm^−1^ for all aged samples. This broadening can be attributed to the formation of hydroperoxide and hydroxylated products of degradation and acid groups formed during the aging of NaCl [51]. The APC coating treated with NC, TC, and UV remained almost unchanged, which means that these coatings are more resistant to aging. This is confirmed by the carbonyl index (CI), that is, the ratio of the absorbances of these peaks at 1728 cm^−1^ and 1227 cm^−1^, respectively. The CI values were calculated according to Formula (1); the values are reported in Table 4. As can be seen, the CI values for coatings aged in the natural environment changed more than slightly for both types of polymer. The CI increased after the coatings were aged in NaCl, so this type of aging demonstrates a significant degradation in their structure. Aging changes the physical and chemical characteristics of the coatings, resulting in rapid color change.

### 3.5. Thermal Properties

**Figure 12:**
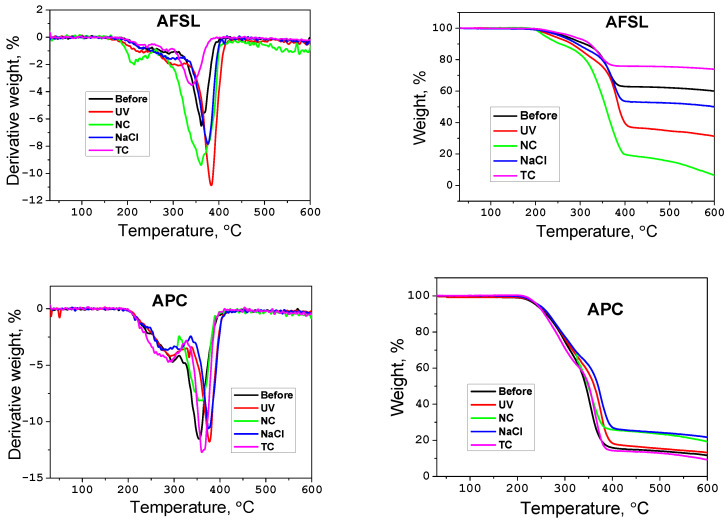
TGA and DTG curves for the coatings.

The difference in the decomposition temperature of the coatings is visible in Figure 12 (TGA and DTG thermograms of the samples) and Table 5 (which summarizes selected parameters from the TGA/DTG thermograms). DTG analysis provides information about the rate of decomposition of samples during the heating process. The coatings decompose in two stages, with the maximum rate of material decomposition occurring around 250 and 350 °C (Table 5, Figure 12).

The TGA curve shows that the thermal stabilities of the AFSL coatings aged by UV radiation or NaCl were initially better than those of the unaged matrix. This is because the delicate chemical bonds and structure were damaged during exposure to ultraviolet light and in the NaCl solution; therefore, the coatings were already very resistant during thermal tests.

When APC coatings were exposed to accelerated ultraviolet aging, aging in the natural environment, and aging in NaCl, the char yield increased, particularly at 600 °C; thermal aging of the sample caused a slight drop in residual mass.

## 4. Conclusions

When the changes in the appearance, microstructure, and adhesion of unaged and aged coatings were analyzed under natural conditions, it was found that despite the relatively short exposure time (five weeks), there were significant changes in the properties of the coatings. The decorative value of all coatings deteriorated significantly due to aging. The changes in color and gloss indicate that oxidation took place. The reduced adhesion significantly reduced the durability of these coatings, thus reducing their reliability and protection against corrosion. The changes in adhesion are reflected in the microstructure of the polymers: the resulting numerous cracks, scratches, and defects indicate that the polymers have degraded, indicating reduced properties of the coatings.

Aging the coatings in NaCl solution had the greatest impact on adhesion. The coatings showing the greatest loss of adhesion showed very little change in their microstructure. Slight visual changes were found during testing, which could signal deterioration of the coating properties. Carbonyl index (CI) values demonstrated significant degradation in their structure during NaCl aging. Therefore, it should be noted that the coating of elements in similar environments may suddenly be damaged without prior warning. Natural aging had a greater effect on the adhesion and thermal stability of the coatings than the influence of UV radiation, but this aging process did not influence the carbonyl index. However, aging through temperature fluctuations did not affect the appearance, microstructure, or wettability of the surface. The main changes were observed for the polyurethane coating, the adhesion of which increased by 74%. This fact is most likely related to the cross-linking of the polymer.

Although the coatings were of the same type (polyurethane–polyurea), some aging factors affect their properties in a slightly different way. Therefore, it should be taken into account in specific applications.

## Figures and Tables

**Table 1:** Data of the resins used in the tests.

	Almacoat Floor SL	Almacoat Protect C
Designation	AFSL	APC
Type of coating	polyurea/polyurethane	polyurea/polyurethane
Density A:B (25 °C), [g/cm^3^]	1.10:1.05	1.13:1.05
Viscosity A:B (25 °C), [mPa∗s]	7000:500	900:650
Mixing ratio A:B (by weight)	100:13	1: 1
Pot life (20 °C) [min]	9	9
Shore hardness	80A	95A
Tensile strength [MPa]	13	19.5
Break elongation (max) [%]	650	495
Stretch resistance (max) [MPa]	13	19.5

**Table 2:** Images used to determine the contact angle θW.

Material	θW [°]	
**AFSL**		
**Before**	82	±1.2
**NC**	74	±1.4
**UV**	40	±3.1
**NaCl**	44	±2.9
**TC**	81	±2.1
**APC**		
**Before**	83	±1.3
**NC**	86	±2.0
**UV**	90	±1.2
**NaCl**	79	±1.9
**TC**	87.5	±2.3

**Table 3:** Surface free energy (SFE) and its components (γsd—dispersive; γsp—polar).

		SFE [mJ/m^2^]	γsd [mJ/m^2^]	γsp [mJ/m^2^]
AFSL	Before	23.66	5.38	18.28
	NaCl	68.74	1.95	66.79
	NC	28.94	8.2	20.75
	TC	24.82	9.15	15.66
	UV	75.39	0.9	74.49
APC	Before	26.86	19.96	6.9
	NaCl	40.61	37.89	2.72
	NC	40.31	37.95	2.36
	TC	20.78	3.33	17.45
	UV	24.84	21.33	3.51

**Table 4:** Carbonyl index of coatings.

		AFSL	APC
**Carbonyl index**	Before	0.36	0.48
	NaCl	1.30	0.71
	NC	0.36	0.48
	TC	0.49	0.45
	UV	0.23	0.48

**Table 5:** Thermal decomposition of the materials.

Material	T_5_ [°C]	T_10_ [°C]	T_50_ [°C]	T_90_ [°C]	R [%]
**AFSL**					
**Before**	240	261	344	641	9.3
**NC**	192	236	349	-	15
**UV**	244	265	362	-	12
**NaCl**	245	265	370	-	20
**TC**	242	257	350	586	6
**APC**					
**Before**	265	310	-	-	58.0
**NC**	221	255	356	563	3.1
**UV**	239	281	386	-	29.7
**NaCl**	256	293	602	-	47.7
**TC**	228	232	-	-	72.3

*T*_5_—the temperature at which the weight loss of the sample reached 5%; *T*_10_—the temperature at which the weight loss of the sample reached 10%; *T*_50_—the temperature at which the weight loss of the sample reached 50%; *T*_90_—the temperature at which the weight loss of the sample reached 90%; *R*—the residual mass.

## Data Availability

Data are contained within the article.

## References

[1] Bordbar S., Rezaeizadeh M., Kavian A. (2020). Improving thermal conductivity and corrosion resistance of polyurea coating on internal tubes of gas heater by nanosilver. Prog. Org. Coat..

[2] Ha S.K., Lee H.K., Kang I.S. (2016). Structural behavior and performance of water pipes rehabilitated with a fast-setting polyurea–urethane lining. Tunn. Undergr. Space Technol..

[3] Rodič P., Cerc Korošec R., Kapun B., Mertelj A., Milošev I. (2020). Acrylate-based hybrid sol-gel coating for corrosion protection of AA7075-T6 in aircraft applications: The effect of copolymerization time. Polymers.

[4] Du Y.J., Damron M., Tang G., Zheng H., Chu C.J., Osborne J.H. (2021). Inorganic/organic hybrid coatings for aircraft aluminum alloy substrates. Prog. Org. Coat..

[5] Yao Y., Sun H., Zhang Y., Yin Z. (2020). Corrosion protection of epoxy coatings containing 2-hydroxyphosphonocarboxylic acid doped polyaniline nanofibers. Prog. Org. Coat..

[6] Zheng S., Bellido-Aguilara D.A., Hu J., Huang Y., Zhao X., Wang Z., Zeng X., Zhang Q., Chen Z. (2019). Waterborne bio-based epoxy coatings for the corrosion protection of metallic substrates. Prog. Org. Coat..

[7] Momber A.W., Plagemann P., Stenzel V. (2016). The adhesion of corrosion protection coating systems for offshore wind power constructions after three years under offshore exposure. Int. J. Adhes. Adhes..

[8] Chen C., Wang X., Hou H., Cheng Y., Zhang P., Liu J. (2022). Effect of strength matching on failure characteristics of polyurea coated thin metal plates under localized air blast loading: Experiment and numerical analysis. Thin-Walled Struct..

[9] Iqbal N., Sharma P.K., Kumar D., Roy P.K. (2018). Protective polyurea coatings for enhanced blast survivability of concrete. Constr. Build. Mater..

[10] Kosiński S., Gonsior M., Krzyżanowski P., Rykowska I. (2021). New Hybrid Polyurea-Polyurethane Elastomers with Antistatic Properties and an Influence of Various Additives on Their Physicochemical Properties. Molecules.

[11] Vaca-Cortes E., Lorenzo M.A., Irsa J.O., Wheat H.G., Carrasquillo R.L. (1998). Adhesion Testing of Epoxy Coating.

[12] Dmitruk A., Mayer P., Pach J. (2017). Pull-off Strength of Thermoplastic Fiber-reinforced Composite Coatings. J. Adhes. Sci. Technol..

[13] Greunz T., Lowe C., Schmid M., Wallner G.M., Strauß B., Stifter D. (2019). Dry adhesion study of polyester/melamine clear coats on galvanized steel. Int. J. Adhes. Adhes..

[14] Gasparin A.L., Wanke C.H., Nunes R.C., Tentardini E.K., Figuero A.C., Baumvol I.J., Oliveira R.V. (2013). An experimental method for the determination of metal–polymer adhesion. Thin Solid Films.

[15] Islama M.S., Tong L., Falzon P.J. (2014). Influence of metal surface preparation on its surface profile, contact angle, surface energy and adhesion with glass fibreprepreg. Int. J. Adhes. Adhes..

[16] Baldan A. (2012). Adhesion phenomena in bonded joints. Int. J. Adhes. Adhes..

[17] Rudawska A. (2014). Selected aspects of the effect of mechanical treatment on surface roughness and adhesive joint strength of steel sheets. Int. J. Adhes. Adhes..

[18] Rudawska A., Danczak I., Müller M., Valasek P. (2016). The effect of sandblasting on surface properties for adhesion, The effect of sandblasting on surface properties for adhesion. Int. J. Adhes. Adhes..

[19] Khan A.A., Abdulaziz A., Kheraif A., Alhijji S.M., Matinlinna J.P. (2016). Effect ofgrit-blasting air pressure on adhesion strength of resin to titanium. Int. J. Adhes. Adhes..

[20] Arikan E., Holtmannspotter J., Zimmer F., Hofmann T. (2019). The role of chemical surface modification for structural adhesive bonding on polymers- Washability of chemical functionalization without reducing adhesion. Int. J. Adhes. Adhes..

[21] Yi J., Boyce M.C., Lee G.F., Balizer E. (2006). Large deformation rate-dependent stress–strain behavior of polyurea and polyurethanes. Polymer.

[22] Whitten I., Youssef G. (2016). The effect of ultraviolet radiation on ultrasonic properties of polyurea. Polym. Degrad. Stab..

[23] Nguyen T.V., Le X.H., Dao P.H., Decker C., Nguyen-Tri P. (2018). Stability of acrylic polyurethane coatings under accelerated and natural outdoor exposure: The critical role of the used photo-stabilizers. Prog. Org. Coat..

[24] Cai G., Wang H., Jiang D., Dong Z. (2018). Degradation of fluorinated polyurethane coating under UVA and salt spray. Part I: Corrosion resistance and morphology. Prog. Org. Coat..

[25] Zubielewicz M., Królikowska A. (2009). The influence of ageing of epoxy coatings on adhesion of polyurethane topcoats and protective properties of coating systems. Prog. Org. Coat..

[26] Pintus V., Wei S., Schreiner M. (2016). Accelerated UV ageing studies of acrylic, alkyd and polyvinyl acetate paints: Influence of inorganic pigments. Microchem. J..

[27] Bhargava S., Kubota M., Lewis R.D., Advani S.G., Prasad A.K., Deitzel J.M. (2015). Ultraviolet, water, and thermal aging studies of waterborne polyurethane elastomer-based high reflectivity coating. Prog. Org. Coat..

[28] Bartolomeo P., Irigoyen M., Aragon E., Frizzi M.A., Perrin P.X. (2001). Dynamic mechanical analysis and Vickers micro hardness correlation for polymer coating UV ageing characterization. Polym. Degrad. Stab..

[29] Królikowska A. (2000). Requirements for paint systems for the steel bridges in Poland. Prog. Org. Coat..

[30] Hu J., Li X., Gao J., Zhao Q. (2009). Ageing behavior of acrylic polyurethane varnish coating in artificial weathering environments. Prog. Org. Coat..

[31] Boubakri A., Guermazi N., Elleuch K., Ayedi H.F. (2010). Study of UV-aging of thermoplastic polyurethane material. Mater. Sci. Eng. A.

[32] Liu P. (2012). Artificial aging test analysis of alkyd and polyurethane coating system. Adv. Mater. Res..

[33] Zhang H., Dun Y., Tang Y., Zuo Y., Zhao X. (2016). Correlation between natural exposure and artificial ageing test for typical marine coating systems. J. Appl. Polym. Sci..

[34] Sanmartín P., Pozo-Antonio J.S. (2020). Weathering of graffiti spray paint on building stones exposed to different types of UV radiation. Constr. Build. Mater..

[35] Che K., Lyu P., Wan F., Ma M. (2019). Investigations on aging behavior and mechanism of polyurea coating in marine atmosphere. Materials.

[36] (2008). Paints and Varnishes—Determination of Film Thickness (ISO 2808:2007).

[37] (2000). Paints and Varnishes—Measurement of Specular Gloss of Non-Metallic Paint Films At 20°, 60°And 85°.

[38] Hanson A.R., Good Practice Guide for the Measurement of Gloss Measurement Good Practice Guide. 2016, 94..

[39] https://www.dataphysics-instruments.com/products/oca/software/#!.

[40] Owens D.K., Wendt R. (1969). Estimation of the Surface Free Energy of Polymers. J. Appl. Polym. Sci..

[41] (2023). Paints and Varnishes, Pull-off Test for Adhesion.

[42] Yu F., Gao J., Liu C., Chen Y., Zhong G., Hodges C., Chen M., Zhang H. (2020). Preparation and UV aging of nano-SiO_2_/fluorinated polyacrylate polyurethane hydrophobic composite coating. Prog. Org. Coat..

[43] Rosu D., Rosu L., Cascaval C.N. (2009). IR-change and yellowing of polyurethane as a result of UV irradiation. Polym. Degrad. Stab..

[44] Huang F., Wei Q., Wang X., Xu W. (2006). Dynamic contact angles and morphology of PP fibres treated with plasma. Polym. Test..

[45] Flandrin F.R., Widmaier J.M., Flat J.J. (1997). Thermal ageing of polyurethane with hydrogenated polyisoprene soft segments. Polym. Degrad. Stab..

[46] Dai Y., Zeng Q. Accelerated Degradation of Coating-mortar Interface under UV Radiation in Presence of Water. Proceedings of the XVI International Conference on Durability of Building Materials and Components DBMC 2023.

[47] Wang W., Feng P., Lv Y., Geng Z., Liu Q., Liu X. (2020). A comparative study on UV degradation of organic coatings for concrete: Structure, adhesion, and protection performance. Prog. Org. Coat..

[48] Arunkumar T., Anish M., Jayaprabakar J., Jeevahan J., Rb D. (2021). Study on mechanical properties of polyurea coating with various process parameters. Mater. Res. Innov..

[49] Phoungtawee P., Crespy D. (2021). Shining a new light on the structure of polyurea/polyurethane materials. Polym. Chem..

[50] Russell J.R., Huang J., Anand P., Kucera K., Sandoval A.G., Dantzler K.W., Hickman D., Jee J., Kimovec F.M., Koppstein D. (2011). Biodegradation of polyester polyurethane by endophytic fungi. Appl. Environ. Microbiol..

[51] Barczewski M., Anisko J., Piasecki A., Biedrzycka K., Moraczewski K., Stepczynska M., Klozinski A., Szostak M., Hahn J. (2011). The accelerated aging impact on polyurea spray-coated composites filled with basalt fibers, basalt powder, and halloysite nanoclay. Compos. B Eng..

